# Results of a phase I, non-randomized study evaluating a Magnetic Occult Lesion Localization Instrument (MOLLI) for excision of non-palpable breast lesions

**DOI:** 10.1007/s10549-019-05499-z

**Published:** 2019-11-21

**Authors:** Nicole Look Hong, Frances C. Wright, Mark Semple, Alexandru M. Nicolae, Ananth Ravi

**Affiliations:** 1 Department of Surgery, Sunnybrook Odette Cancer Centre, Toronto, ON M4N-3M5 Canada; 2 Department of Medical Physics, Sunnybrook Odette Cancer Centre, 2075 Bayview Ave, Toronto, ON M4N-3M5 Canada; 3 Clinical Research Program, Sunnybrook Research Institute, Toronto, ON M4N-3M5 Canada

**Keywords:** Lumpectomy, Breast cancer, Localization, Guidance

## Abstract

**Purpose:**

Magnetic Occult Lesion Localization Instrument (MOLLI) is a wireless, non-radioactive alternative for non-palpable breast lesion localization. The primary objective of this first-in-human study was to evaluate the clinical feasibility of using MOLLI for intraoperative localization of non-palpable breast lesions.

**Methods:**

Twenty women with non-palpable breast lesions at a single institution received a lumpectomy using the MOLLI guidance system. Patients were co-localized with magnetic and radioactive markers up to 7 days before excision by a dedicated breast radiologist under sonographic guidance. Both markers were localized intraoperatively using dedicated hand-held probes. The primary outcome was successful excision of the magnetic marker, confirmed radiographically and pathologically. Demographic data, margin positivity, and re-excision rates were collected. Surgical oncologists, radiologists, and pathology staff were surveyed for user satisfaction.

**Results:**

*Post-radiological analysis:* Post-implant mammograms verified that 17/20 markers were placed directly in the lesion center. Radiologists reported that all marker implantations procedures were “easy” or “very easy” following a single training session. *Post-surgical analysis:* All MOLLI markers were successfully removed with the specimen during surgical excision. In all cases, surgeons ranked the MOLLI guidance system as “very easy” for lesion localization. *Pathologic analysis:* All patients had negative margins. All anatomic pathology staff ranked the MOLLI system as “very easy” to localize markers.

**Conclusions:**

The MOLLI guidance system is a reliable and accurate method for intraoperative localization of non-palpable breast lesions. Further evaluation of the MOLLI system in studies against current standards of care is required to demonstrate system cost-effectiveness and improved patient-reported outcomes.

## Introduction

As many as 60% of all diagnosed breast cancers are non-palpable; therefore, accurate pre-operative localization of these lesions plays a pivotal role in guiding surgical excision [1]. The two most established techniques for pre-operative localization of breast lesions are wire localization (WL) and radioactive seed localization (RSL). WL involves percutaneous implantation of a hooked wire under image guidance to mark the center or outer edges of target lesions [2]. WL, although widely adopted has disadvantages which include possible wire displacement [3], patient discomfort, and poor workflow efficiency, as the wire implantation is typically scheduled the same day as the surgery [3]. RSL involves implanting a small radioactive seed to identify the lesion and/or its borders [4]. The surgeon uses a hand-held probe to localize the seed. The primary advantage of RSL compared to WL is the improved patient experience as there is no longer a wire extending outside the breast. Additionally, RSL enables increased flexibility and efficiency of scheduling, as seeds can be placed days to weeks prior to surgical excision. Additionally, seeds provide a much more focal target, thus optimizing excisional accuracy. While RSL addresses many issues associated with WL, the radioactive seeds introduce significant regulatory and radiation safety requirements that add to procedural cost and complexity [5].

**Fig. 1:**
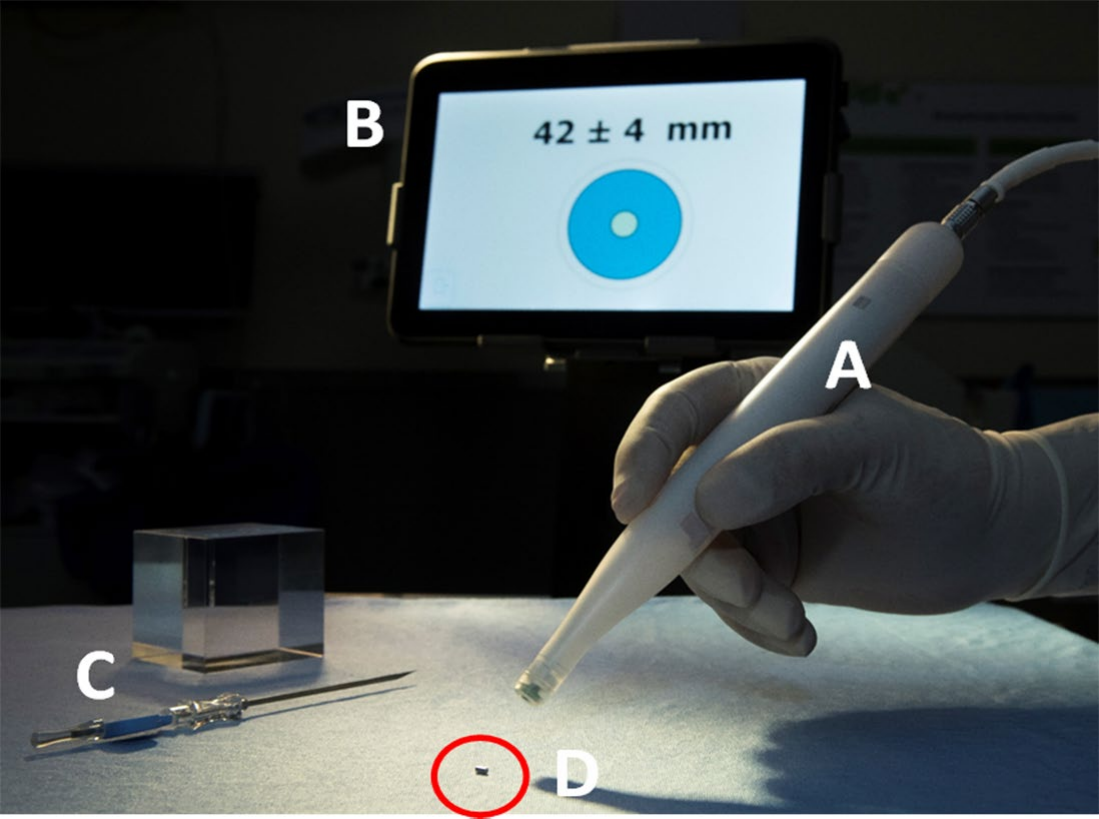
Display of the MOLLI system showing **a** the hand-held detector probe, **b** the tablet PC displaying the graphical and audio feedback system, **c** the marker introducer, and **d** the implantable marker (highlighted in the red circle)

In 2016, the Sunnybrook Odette Cancer Centre developed a non-radioactive wireless localization system as an alternative to both WL and RSL called the Magnetic Occult Lesion Localization Instrument (MOLLI). MOLLI was designed with accuracy, ease-of-use, and cost-effectiveness in mind (Fig. 1). The system, analogous to RSL, implants a small magnetic marker directly in or around the breast lesion. A purpose-built probe is used by the surgeon to measure the distance from the skin to the implanted marker/lesion; the distance is then displayed on a tablet computer in addition to an auditory and visual feedback system. In 2017–2018, pre-clinical characterization studies of the system showed promise for using MOLLI in the context of localization procedures [6]. Reliable marker detection up to 53 ± 8.56 mm deep to the tissue surface was achieved, and bracketing with markers spaced as close as 10 mm apart (at 42 mm depth) was demonstrated. Finally, metallic surgical instruments and conventional operating room environments did not affect the magnetic localization process, system accuracy or reliability [6]. With this background of promising pre-clinical parameters, this study aims to evaluate the safety and efficacy of MOLLI in a phase I, non-randomized study, first-in-human study.

## Methods

### Study population

Female patients > 18 years old with high-risk premalignant or malignant lesions eligible for breast conserving surgery were accrued into the study. Lesions were required to be non-palpable, unifocal, and localizable by ultrasound. Patients were identified in surgical oncology clinics and consented by study personnel. Research ethics board approval was obtained at Sunnybrook Health Sciences Centre.

### Intervention – use of MOLLI guidance system

#### Radiology

Patients participating in the study all received concurrent implantation of both MOLLI and RSL (Isoaid, FL, USA) markers under sonographic guidance. Patients were locally anesthetized preceding the RSL seed implantation in the lesion. Subsequently an 18G, 7 cm long MOLLI introducer needle was used to implant the custom-made MOLLI marker (1.6 mm diameter × 3.8 mm in length, Fig. 1) as close as possible to the RSL seed. Seven breast radiologists with expertise in breast imaging performed the implantation procedures. Total implant time was recorded. Orthogonal mammograms were then obtained following the procedure to confirm successful placement of both markers, as well as validation of any MOLLI marker migration relative to the RSL seed [7].

#### Surgery

Surgery was scheduled up to 7 days after initial implantation. Both MOLLI and RSL probes (Neoprobe, Leica Biosystems, Germany) were set up in the operating room. The MOLLI probe is sterilizable using Sterrad® or ethylene oxide; however, as an additional precaution, both the RSL and MOLLI the probes were draped in individual sterile sheaths before the procedure. The surgeon used both the MOLLI probe and RSL probe to localize the respective markers. Data on the depth of the marker to the skin surface were recorded for the MOLLI marker. Once the specimen was excised, the MOLLI probe was used to confirm the removal of the magnetic marker with the specimen. Total operative time was recorded, measured as the time from the patient sedation to the complete removal of the specimen from the breast cavity. Post-surgical radiographic imaging (Faxitron, Hologic, Malrborough, MA, US) was taken to confirm both markers were present in the specimen. Two breast surgeons performed all of the surgeries.

#### Pathology

Immediately following the surgery, the excised specimen was transported to pathology for initial margin evaluation and removal of the implanted markers. The MOLLI marker was removed from the sample and discarded in standard bio-hazardous waste. RSL seeds were removed and stored according to local protocols.

#### Outcomes

The primary outcome was to evaluate whether the MOLLI marker could be successfully localized and removed from all patients, and to ensure that there were no adverse events as a result of its use. Secondary outcomes included changes in patient-reported quality-of-life outcomes, margin status, re-excision rates, procedural time and physician satisfaction with the use of the MOLLI system. Patient-reported quality of life was measured at day 0 prior to implantation and at day 30 after implantation, using the validated EQ 5D-3L and EQ-VAS questionnaires [8, 9]. Similarly, breast radiologists, breast surgeons and pathology assistants were asked to complete a short five-point Likert scale questionnaire to evaluate their experience using MOLLI immediately after their interaction with the system, or completion of their respective procedure.

### Statistical analysis

Patient and lesion characteristics were collected prospectively at the time of patient enrollment. Procedural and pathological data were collected from patient electronic medical records once surgical pathology was available. Physician satisfaction surveys were completed on the day of the respective procedure. Equation 5D-3L and EQ-VAS questionnaires were obtained at day 30 following initial implantation by phone follow-up. Generalized descriptive statistics were reported for all study outcomes. Categorical variables are reported as counts, percentages, or proportions while continuous variables are represented as means, ranges, and 95% confidence intervals. Statistical analysis was conducted using Python v3.6 and the SciPy v9.0 statistics package.

## Results

Patient demographics and tumor characteristics (following pathological analysis) are listed in Table 1. Study outcomes are reported in Table 2. Overall total radiological time (to insert both seeds) and operative times were mean 11.85 ± 6.81 and 36.3 ± 16.6 min, respectively. MOLLI markers were successfully placed in all cases by seven different breast radiologists. Additionally, all of the MOLLI markers were localized prior to incision at an average depth of 13.6 ± 5.22 mm. Negative margins were achieved in all cases with no patients requiring re-excision. There were no adverse events or complications reported as a result of the implantation or the surgery.

**Table 1:** Patient demographics and tumor characteristics

	Average ± SE (*n* = 20)
Age (years)	60.3 ± 13.0
Height (m)	1.6 ± 0.1
Weight (kg)	66.7 ± 12.6
Menopausal status	
Pre	6
Post	14
Tumor type	
DCIS	3
IDC	12
Other	5
Receptor status	
ER/PR+, HER2−	10
ER−/PR+, HER2−	1
ER/PR−, HER2−	1
Not evaluated^a^	8
Largest tumor size (mm on imaging)	15.7 ± 8.7
BIRADS category	
5	10
4	10
Tumor stage	
p2a	4
p1a	8
p0	5
Not applicable	3
Tumor grade	
3	5
2	8
1	4
Not evaluated	3
Multifocal disease	
Yes	1
No	19
Lymph node metastases	
Yes	3
No	9
Not evaluated	8

*BMI* body mass index, *DCIS* ductal carcinoma in situ, *ER* estrogen receptor, *IDC* invasive ductal carcinoma, *PR* progesterone receptor, *SE* standard error

^a^Not evaluated category for DCIS or other receptor status

**Table 2:** Study outcomes

Radiology	
Days prior to surgical procedure; mean (range)	1.25 (0–7)
Number of radiologists	7
Marker placed in lesion center	17
Marker successfully placed	20
Total procedure time; mean (range)	11.85 (– 35) min
Surgery	
Number of surgeons	2
Marker found before incision	20
Marker depth from skin; mean (range)	13.6 (8–29) mm
Marker successfully removed	20
Marker in lesion center	13
Marker in specimen	20
RSL needed to confirm position	2
Total operative time; mean (range)	36.3 (21–80) min
Pathology	
Number of pathologists	6
Negative margins	20
Marker removed	20
Re-excision required	0

*RSL* radioactive seed localization

**Fig. 2:**
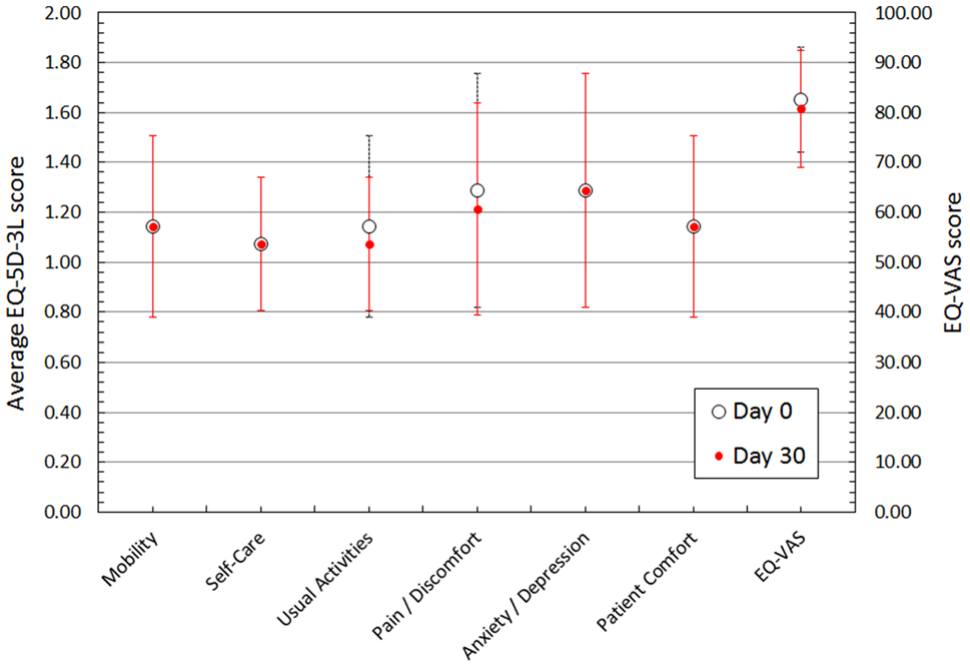
Patient-reported outcomes at day 0 and day 30 following implantation with the MOLLI marker. There were no significant value changes between day 0 and day 30. EQ-5D-3L scoring: 1 = no problems, 2 = some problems, 3 = severe problems. EQ-VAS scoring: 1 = worst imaginable health to 100 = best imaginable health. Dashed lines represent error bars for day 30 outcomes and solid lines represent error bars for day 0 outcomes

PROs (EQ-5D-3L and EQ-VAS) at day 0 and day 30 are reported in Fig. 2. No significant differences were observed between day 0 and day 30 outcomes both overall and when stratified by confounding factors (age, BMI, menopausal status, tumor size). Day 30 reported outcomes were worse than day 0 although these were not significant overall.

**Fig. 3:**
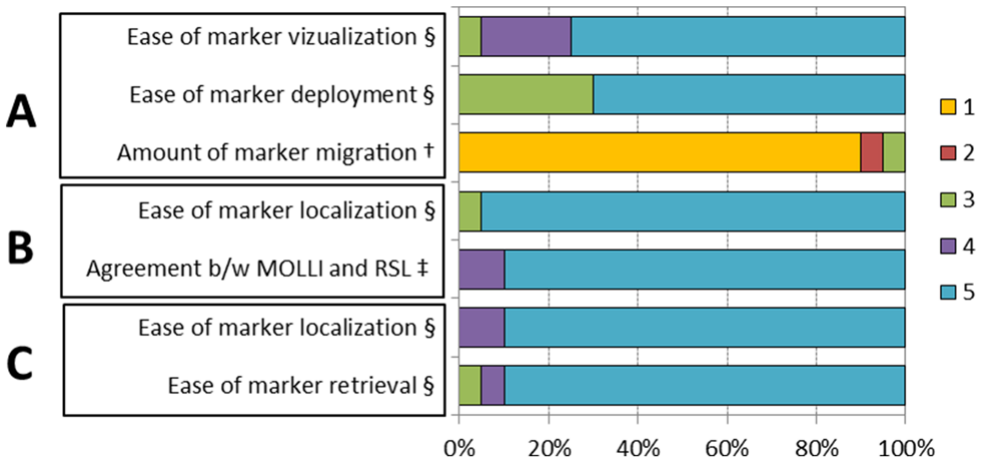
Physician experience with the MOLLI system. Physician-reported satisfaction surveys showing **a** radiology, **b** surgery, and **c** pathology responses. Results are reported as a percentage of total respondents for each category: ^**§**^1 = very hard, 3 = neutral, 5 = very easy, ^**†**^1 = none, 3 = some, 5 = significant, ^**‡**^1 = poor, 3 = neutral, 5 = excellent

Physician satisfaction survey results are presented in Fig. 3. The majority of physicians reported “easy” or “very easy” marker visualization (radiology), deployment (radiology), and localization (surgery and pathology). Physicians reported an overall “excellent” agreement between MOLLI and RSL markers intraoperatively with no MOLLI marker migration.

## Discussion

This study represents the first-in-human evaluation of the novel MOLLI technology. Pre-clinical benchmarking studies of MOLLI demonstrated significant benefits over RSL including a direct measurement of the distance from the probe to the marker with a visual and audio feedback system to assist surgeons with accurate localization. Depth of detection of up to 53 mm was achieved, allowing verification of suspicious lesions deep to the skin surface. The results of the current clinical trial confirm pre-clinical findings that MOLLI is a feasible intraoperative guidance system. Physician-reported survey responses and PROs at day 30 were promising, and all 20 patients had negative margins on follow-up.

The current dominant localization techniques of RSL and WL are clinically effective and well tolerated. There is equipoise in terms of clinically significant measures such as margin positivity rates and volume resected. As such, selection of localization technology is likely going to be a multifactorial decision based on patient satisfaction, clinician usability, and health system effectiveness [10].

MOLLI is a beneficial addition to the rapidly developing area of breast localization technologies. Other products use technologies that have been adapted from alternative, typically non-medical, applications to suit breast lesion localization. MOLLI has been designed to overcome many of the challenges faced by some of these emerging technologies, as reported in literature, such as: MagSeed (Endomag, UK) which is sensitive to metallic surgical instruments, is limited in depth of detection to 4 cm, and cannot measure marker distance [11] [12]; Scout® (Merit Medical, US) which has a high cost and issues with marker resection; and LOCalizer (Faxitron, US) which is encumbered by blind spots in detecting the marker [13, 14]. However, clinical evaluation of the comparative benefits of these localization technologies is still required in a future study. The MOLLI system was specifically designed for wireless breast lesion localization, without using radiation, and uses simple, sterilizable, cost-effective technology that dramatically reduces the complexity and human resource requirements of localization procedures.

### Physician-reported outcomes

Physician survey outcomes showed generally favorable reviews of the MOLLI approach (Fig. 3). In terms of marker deployment, ease of visualization on ultrasound, and ease of localization, the majority of physicians ranked these tasks as “easy” or “very easy.” Physicians familiar with RSL required a single training session to be comfortable using the MOLLI introducer and system. Migration of magnetic markers was also reported as not occurring or “minimal” on par with the much larger RSL marker [7]. The spatial localization of the MOLLI marker compared to RSL was reported be “excellent” between the two was reported by surgeons. There was no interference reported from standard metal instruments or the Technecium-99m sulfur colloid used for sentinel lymph node biopsy. Finally, the retrieval and disposal of the magnetic marker was reported “very easy” by pathologists.

### Patient-reported outcomes

Decreases in overall mean EQ-5D-3L and EQ-VAS scores were reported between day 0 and day 30, although these were not significant changes (Fig. 3). Similar overall mean decreases were reported when patients were stratified by potential confounding variables. Overall decreases, although not significant, may represent decreases in quality of life related to undergoing a surgical intervention under anesthesia and are likely the result of normal fatigue and discomfort associated with the surgical procedure.

### Study outcomes and pathological analysis

Radiologists adopted the MOLLI system after just one training session, speaking to the ease-of-use. Radiologists were able to place the MOLLI marker in the lesion center using ultrasound guidance and the MOLLI introducer in 17 (85%) of cases (Table 2). An average procedure time was only 11.85 ± 6.81 min. The total implant time may be significantly shorter, as RSL markers were concurrently implanted with MOLLI markers. During the surgery, all of the MOLLI markers were found prior to the actual excision, and all were successfully removed and captured within the excised specimen. The average depth of implanted MOLLI markers was 13.6 ± 5.22 (8–29) mm as measured using the MOLLI probe. Total operative time was on average 36 ± 17 min, including time from anesthetic administration, using both RSL and MOLLI components to the point of excision of the specimen. Following pathological analysis, all patients had negative surgical margins and did not require re-excision indicating the functional ability of the MOLLI technology to assist surgeons with adequate lesion localization in the intraoperative setting.

### Future direction

The study was limited to a single institution with experience in the RSL workflow and with seed-localized lumpectomies, which likely contributed to the quick uptake of the MOLLI technology. After initial success with the first 20 patients, a larger scale multi-center registry study has been submitted for ethics review at the time of manuscript submission to evaluate the MOLLI system further. The registry study is expected to obtain further evidence of efficacy, obstacles to adoption, and cost-effectiveness data. Aggregate data from this study are expected to demonstrate the utility of MOLLI across institutions and various types of health care systems for breast localization procedures. MOLLI is currently being commercialized by (Toronto, ON, Canada) and is expected to receive FDA 510(K) clearance by September 2019.

## Conclusion

This study demonstrates the safety and feasibility of using the MOLLI technology within the intraoperative setting to localize non-palpable breast lesions. Radiologists, surgeons, and pathologists reported quick uptake and satisfaction with the procedure. Finally, all 20 (100%) patients had successful identification and removal of the MOLLI marker with the excised specimen, and no patients required re-operation for positive surgical margins on pathological analysis. The findings of this study are encouraging but demonstrate the need for more comprehensive comparative evaluation to build an evidence base for the MOLLI technology in the setting of lesion-localized lumpectomies.
